# The Pleasures of Imagination Defended

**Published:** 1889-09-07

**Authors:** 


### The Pleasures of Imagination Defended.

The scientific uses of the imagination have been ere this the theme of the philosopher, and its abuses the subject of the sermon of the preacher, but never before have so many brilliant things been said in defence of the powers and pleasures of imagination as by Sir James Crichton Browne in his address to the British Medical Association, which has just met at Leeds. "All true science rests on imaginings," he boldly asserts, and this statement he elucidates by showing how imagination's help is called in to supply scientific men with speculations and hypotheses, to trace disease to its source, and to arrange, combine, and co-ordinate the isolated facts of knowledge. But besides availing themselves of the uses, Sir Crichton urges his medical brethren to enjoy the pleasures of the imagination, and to "ramble for a little in the green pastures of literature or climb the pinnacles of art." He looks on undismayed at the growing taste for the literature of fiction, as shown by the returns from the lending libraries, inasmuch as he asserts that as men become more educated the influence of tales of horror and crime is not to be dreaded; for it is only the ignorant and neglected boy, keen, perhaps, with that superficial sharpness that is really a kind of imbecility, but with a narrow intellectual horizon, that is lured into wickedness by the penny dreadfuls; and the silly and dissipated youths of better social position who go to ruin can rarely trace their downfall to poetry or fiction; while to the healthy minded, imaginative literature may heighten happiness, afford solace in suffering and sorrow, give brace to exertion, and lessen the sense of fatigue. It is a mistake, Sir Crichton asserts, to think that indulging in the pleasures of imagination induces insanity, for "for one case of insanity caused by excess of imagination there are a dozen caused by want of it." The insane are the least imaginative of beings, the lunatic is as dull as a stone, and idiocy is the absolute negation of imagination. One of the most valuable aids in the treatment of the insane is the imagination, which is invoked in every possible way, by music, pictures, and the drama in all lunatic asylums worthy of the name, and the first recorded case of the cure of melancholia was by the harp of David. Religion, which the Bishop of Ripon said had been accused of being "the nurse of hysteria and the mother of hallucinations," Sir Crichton Browne declares "to make for sanity," by affording support under sorrow and misfortune, maintaining mental equilibrium in agitating times, and supplying lofty motives to conduct, and so cleansing and controlling life; but on faith-healers and the "peculiar people" the orator pours his sarcasm, and calls upon them to learn how to make an accurate clinical report, and to apprehend what induction means, before assertin the cure of diseases which were simulated, or which ran a natural course or were purely subjective. Here is an example of imagination run wild, but its normal use in the economy might be spoken of as that of a pioneer opening up new pathways in the brain, or as a mode of transit between its territories. The brain that is without imagination is like a country without roads or railways, in which locomotion is laborious and slow; and the brain richly gifted with it is like one in which steam and electricity have established easy and rapid communications. "If," as Matthew Arnold says, "two-thirds of life are conduct," it may truly be alleged that three-fourths of thought are imagination. It is not merely the child who builds castles in the air. Each of us—the most matter-of-fact as well as the most romantic—has "cloud-capped palaces and gorgeous towers," known to himself alone, to which, in hours of solitude and reverie, or sadness and chagrin, he retires with ever-renewed wonder and exaltation, and from which he scatters beneficence on all around. And the great faculty that out of thin air constructs these cities of refuge for us, and enables us, in a sense, to shape our own environments, is the handmaid of science, the giver of insight, and the harmoniser of discords.

We must add our grateful thanks to Sir Crichton Browne for a defence so eloquent and scientific of one of the greatest pleasures of life.

